# In Situ Synthesis of Hierarchical Co(CO_3_)_0.5_(OH)·0.11H_2_O@ZIF‐67/WO_3_ With High Humidity Immunity and Response to H_2_S Sensing

**DOI:** 10.1002/advs.202402352

**Published:** 2024-08-19

**Authors:** Yanghai Gui, Jintao Wu, Di Zhao, Kuan Tian, Shuaishuai Zhao, Huishi Guo, Xiaoyun Qin, Xiaomei Qin, Dongjie Guo, Yun Wang

**Affiliations:** ^1^ College of Materials and Chemical Engineering Zhengzhou University of Light Industry Zhengzhou 450000 China; ^2^ Centre for Catalysis and Clean Energy School of Environment and Science Griffith University Gold Coast QLD 4222 Australia

**Keywords:** Co(CO_3_)_0.5_(OH)·0.11H_2_O@ZIF‐67/WO_3_, gas‐sensing, H_2_S, in situ growth, mechanism analysis

## Abstract

H_2_S gas sensors with facile preparation, low detection limits, and high selectivity are crucial for environmental and human health monitoring. However, it is difficult to maintain a high response of H_2_S gas sensors under high humidity in practical applications. To face this dilemma, a layer‐by‐layer growth method is applied to in situ prepare a nanostructured Co(CO_3_)_0.5_(OH)·0.11H_2_O/WO_3_ coated by a hydrophobic hierarchical ZIF‐67 as the H_2_S sensor. This novel composite exhibits excellent humidity immunity without sacrificing the excellent sensitivity and selectivity of H_2_S. At a low operating temperature of 90 °C, a remarkable response value of 1052.3 to 100 ppm H_2_S has been achieved, which is 779 and 9.36 times higher than that of pure WO_3_ and Co(CO_3_)_0.5_(OH)·0.11H_2_O/WO_3_, respectively. More importantly, an 82.2% relative response value remains at a high humidity of 75%RH. The sensing mechanisms are investigated using gas chromatography‐mass spectrometry (GC‐MS), which revealed that the reaction products are H_2_O and SO_2_. The high humidity immunity and fast response of the Co(CO_3_)_0.5_(OH)·0.11H_2_O@ZIF‐67/WO_3_ demonstrate the layer‐by‐layer in situ synthesis method holds the potential application for the development of high‐performance WO_3_‐based H_2_S sensors.

## Graphical abstract

A novel in situ layer‐by‐layer growth method is developed to synthesize core‐shell structured Co(CO_3_)_0.5_·0.11H_2_O @ZIF‐67/WO_3_ composites, which exhibit remarkable H_2_S gas sensing performance, as evidenced by high selectivity and response, and a low detection limit. More importantly, it has excellent resistance to high humidity, making it a promising candidate for practical H_2_S sensing applications in environments where humidity is a concern.

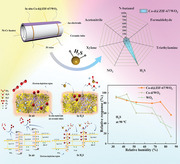

## 1 Introduction

Hydrogen sulfide (H_2_S) is a highly toxic and pervasive gas in industries, which poses severe health and environmental risks from leaks and emissions. High concentrations of it are lethal on minimal exposure.^[^ ^1^, ^2^ ^]^ H_2_S also serves as a biomarker for asthma and chronic obstructive pulmonary disease, rendering its detection in exhaled breath crucial for early disease diagnosis and real‐time health monitoring.^[^ ^3^ ^]^ Therefore, the development of H_2_S gas sensors with a low detection limit and high selectivity through facile preparation methods holds a significant practical implication for effective environmental and human health monitoring.

Metal oxide semiconductors (MOS), such as WO_3_,^[^ ^4^ ^]^ SnO_2_,^[^ ^5^ ^]^ and ZnO,^[^ ^6^ ^]^ have been widely used to detect toxic gases due to their unique electronic properties and thermal stability. Specifically, WO_3_ has gained significant attention in gas‐sensing research since it is an n‐type semiconductor material with a wide bandgap (2.6–3.2 eV).^[^ ^7^ ^]^ However, the practical applications of WO_3_‐based sensors are limited by their inadequate humidity immunity. Humidity is an ineluctable factor in sensor tests, which exerts a significant influence on the performance and stability of sensors, such as signal drift, as well as sensitivity and selectivity degradation.^[^ ^8^, ^9^ ^]^ Therefore, improving the humidity immunity becomes important to their practical applications.

In previous studies, Gao et al. coated a PDMS hydrophobic film on TiO_2_‐based H_2_ sensors via thermal evaporation to enhance humidity protection.^[^ ^10^ ^]^ Nonetheless, adding humidity barriers frequently diminishes sensor sensitivity by inhibiting molecule‐sensor interactions,^[^ ^11^ ^]^ necessitating the development of a method to enable humidity immunity of sensors without compromising their sensing efficacy. An appealing strategy is to incorporate hydrophobic porous materials, like metal‐organic frameworks (MOFs), in sensors to prevent moisture interference.^[^ ^12^, ^13^, ^14^ ^]^ For example, Qin et al. employed the highly humidity‐resistant ZIFs to boost the moisture tolerance of gas sensors and enable the detection of minute ethanol quantities.^[^ ^15^, ^16^, ^17^ ^]^ However, the direct incorporation of MOFs with sensors can yield large or misshapen crystals, impairing sensing efficiency.^[^ ^18^ ^]^ An alternative method is to form MOF shells on metal oxide surfaces through ligand‐assisted extraction of metal ions. The feasibility of this method has been demonstrated by Liu et al., who used this approach to synthesize ZnO@ZIF‐8 with enhanced ethanol detection.^[^ ^19^ ^]^ Luo et al. and Cai et al. further exemplify this approach with customized ZnO/Pd@ZIF‐8 and CoO@ZIF‐67 composites, respectively, emphasizing the significance of optimal reaction conditions and precursor selection for successful and uniform MOFs growth.^[^ ^20^, ^21^ ^]^

Stimulated by these recent experiments, we propose to use a ZIF‐67 epitaxial film as humidity immunity material to be seamlessly integrated with the WO_3_‐based H_2_S sensor. Conventional fabrication techniques for gas sensors, including spin‐coating, inkjet printing, and impregnation, grapple with material loss, uneven dispersion, and compromised uniformity issues. Consequently, they are not suitable for preparing this integrated composite.^[^ ^22^, ^23^, ^24^ ^]^ As an alternative, the in situ growth method can efficiently circumvent these obstacles to assure integrity without cracks, powder dispersion, or inconsistent coating. In this regard, the in situ growth of MOFs directly on the Co(CO_3_)_0.5_(OH)∙0.11H_2_O/WO_3_ heterojunction is employed in this study. Co(CO_3_)_0.5_(OH)∙0.11H_2_O (referred to as Co‐d) is selected due to its facile synthesis process, eco‐friendliness, affordability, high surface area, and spacious 3D structure.^[^ ^25^, ^26^, ^27^, ^28^, ^29^ ^]^ Our results demonstrate that Co‐d@ZIF‐67/WO_3_ composites through in situ growth of ZIF‐67 from Co‐d on WO_3_ have a substantial enhancement of the H_2_S gas‐sensing performance. Compared to the Co‐d/WO_3_ sensor, the response value of the Co‐d@ZIF‐67/WO_3_ sensor increases by 9.38 times to 100 ppm H_2_S at 90 °C. Most importantly, their humidity immunity has been significantly improved.

## 2 Experimental Section

### 2.1 Chemical Reagents

Tungsten hexachloride (WCl_6_, Aladdin Co., Ltd.), ethanol (Tianjin Fuyu Fine Chemical Co., Ltd.), cobalt nitrate hexahydrate (Co(NO_3_)_2_·6H_2_O, Tianjin Komeo Chemical Reagent Co., Ltd.), Polyethylene oxide‐polypropylene oxide‐polyethylene oxide (PEO_20_‐PPO_70_‐PEO_20_, P123, Sigma‐Aldrich Trading Co., Ltd), urea (CH_4_N_2_O, Tianjin Dern Chemical Reagent Co., Ltd.), 2‐methylimidazole (Tianjin Komeo Chemical Reagent Co., Ltd.), *N*,*N*‐Dimethylformamide (DMF, Sinopharm Chemical Reagent Co., Ltd.), and deionized water. The chemicals for the experiments were all analytically pure without further purification.

### 2.2 Preparation of WO_3_

The synthesis of WO_3_ was based on the previous work.^[^ ^30^ ^]^ Briefly, P123 (0.1833 g) was dissolved in a mixture of distilled water (0.25 mL) and ethanol (28.00 mL). WCl_6_ (0.7205 g) was added under stirring conditions until the solution was bright yellow. The cleaned ceramic tube was immersed in the solution for 2 min and removed to dry, and this process was repeated three times. Subsequently, the ceramic tube was suspended in the 50 mL Teflon‐lined stainless‐steel autoclave with the solution and hydrothermally reacted at 150 °C for 3 h. Thereafter, the ceramic tubes with in situ grown WO_3_ nanosheets were rinsed with distilled water and ethanol several times and dried at 50 °C. Eventually, the tubes were calcined at 400 °C for 2 h at a rate of 1 °C min^−1^.

### 2.3 Preparation of Co‐d/WO_3_

A solution was prepared by dissolving Co(NO_3_)_2_·6H_2_O (2.1807 g) in deionized water (50.00 mL) under stirring and ultrasonication until complete dissolution. The ceramic tube with WO_3_ nanosheet was immersed in a sequential process of moistening and dried in the solution for three cycles. Subsequently, urea (3.2906 g) was introduced to the solution and stirred for 5 min until complete dissolution. The WO_3_ ceramic tubes were immersed in the resulting solution and subjected to a reaction at 90 °C in a water bath for 20 min, leading to the in situ growth of Co‐d/WO_3_ material.

### 2.4 Preparation of Co‐d@ZIF‐67/WO_3_

**Figure 1:**
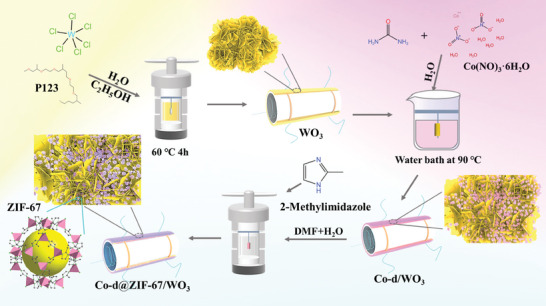
Schematic of the experimental process of Co‐d@ZIF‐67/WO_3_ nanomaterial.

2‐methylimidazole (0.0408 g) was dissolved in a mixed solution of deionized water (4.00 mL) with DMF (12.00 mL) under stirring conditions and sonicated until completely dissolved. The above Co‐d/WO_3_ ceramic tubes were immersed in the mixed solution and placed into a 50 mL Teflon autoclave for hydrothermal reactions at 60 °C for 2, 4, and 8 h, respectively. The obtained ceramic tubes were cleaned in DMF and dried, which were named as Co‐d@ZIF‐67/WO_3_‐2, Co‐d@ZIF‐67/WO_3_‐4, Co‐d@ZIF‐67/WO_3_‐8, respectively. The experimental process diagram of Co‐d@ZIF‐67/WO_3_ nanomaterial is shown in **Figure** **1**.

### 2.5 Characterizations

X‐ray diffraction (XRD) pattern was obtained by using a Bruker D8 instrument with a Ni‐filtered Cu‐Kα1 radiation (λ = 0.154 056 nm). High‐resolution Transmission Electron Microscopy (HRTEM) was performed on a JEOL JSN‐2100 F instrument. Field Emission Scanning Electron Microscope (FE‐SEM) was performed on a JEOL JSM‐7001F SEM apparatus. The FT‐IR spectra of the prepared samples were collected by using a Thermo Scientific Nicolet 6700 instrument with a diamond‐attenuated total reflection module in the frequency range of 400–4000 cm^−1^. Gas Chromatography‐Mass Spectrometer (GC‐MS) (ISQ) was used for gas‐sensing mechanism analysis.

### 2.6 Gas‐Sensing Performance Test

The diagram of the gas sensor structure with the corresponding test system circuit is presented in Figure S1 (Supporting Information). The ceramic tubes with the in situ grown materials were soldered to a hexagonal base and heated using a Ni‐Cr heating wire to achieve the necessary operating temperature. After aging the components at room temperature for 3 days, the soldered components were connected to the test board. The WS‐30A test system (Zhengzhou Weisheng Electronic Technology Co., Ltd.) was used to evaluate their gas‐sensing performance. The specific test process is shown in Figure S2 (Supporting Information). The heating voltage (V_h_) was adjusted to control the working temperature of the ceramic tube from room temperature to 120 °C. The real‐time detection of resistance changes was implemented by computer. After 60 s of baseline stabilization, the gas was injected into the closed test chamber (different gases of the same concentration for selective testing and different concentrations of the same gas for different concentration testing). For VOC gases, the liquid was injected on a high‐temperature evaporation platform to vaporize rapidly. The target gas was diffused by the high‐speed fan in the detection box. The concentration to be measured was reached in a very short time. Subsequently, after the reaction in the box was stabilized with constant resistance, the elements were exposed to the air at a uniform time to be recovered. The resistance changes of the elements were continuously detected. The test process ended when the component resistance returned to the initial state. The selectivity of the sensor to different gases was reflected by their response values.

The gas‐sensing performance was tested based on the change in resistance value of the component in response to either air or the target gas. Specifically, we define the gas response value as *S_r_* = *R_a_*/*R_g_* (for reducing gases) or *R_g_*/*R_a_* (for oxidizing gases), where *R_a_* represents the resistance in the air atmosphere and *R_g_* is the resistance in the target gas atmosphere. Additionally, the response time (T*_res_*) and recovery time (T*_rec_*) were measured, which indicates how quickly the resistance of sensors changes to 90% of its maximum value upon gas addition or removal.^[^ ^31^ ^]^ The humidity environment of the gas mixture was established by utilizing saturated solutions of inorganic salts. The preparation process involved generating relative humidity (RH) levels of 33%, 43%, 52%, 67%, 75%, and 85% using saturated solutions of MgCl_2_, K_2_CO_3_, Mg(NO_3_)_2_, CuCl_2_, NaCl, and KCl, respectively.^[^ ^32^, ^33^ ^]^

### 2.7 Gas‐Sensing Mechanism Analysis

The Gas Chromatography‐Mass Spectrometry (GC‐MS) was used to understand the gas‐sensing mechanism. Prior to the injection, all sample testing conditions were set at 90 °C for 1 h, with careful control over the injection volume, injection depth, and gas injection amount. The specific procedure was as follows: 1) air samples were tested in headspace vials as controls; 2) H_2_S (1.00 mL) was injected into the headspace vial, which was then shaken at 90 °C for 1 h before testing; and 3) ceramic tube with in situ grown Co‐d@ZIF‐67/WO_3_ was placed at the bottom of the headspace vial and injected with 1.00 mL of H_2_S under the same reaction conditions as mentioned above. The obtained gas chromatography and mass spectrometry graphs of different samples were compared to explore the gas‐sensing reaction mechanism of the materials.

### 2.8 Statistical Analysis

The limit of detection (LOD) of the sensor is calculated as the following equation:

(1) LOD=3×σ/S

where σ is the standard deviation of the test baseline based on its data points of the selected sensor (N = 60), and S is the slope of the concentration‐response value curve. The 60 baseline resistance data are listed in Table S1 (Supporting Information). Microsoft Excel software was used for data processing.

## 3 Results and Discussion

### 3.1 Structure and Morphology

**Figure 2:**
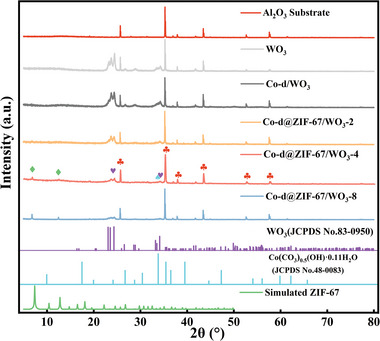
XRD patterns of Al_2_O_3_ substrate, WO_3_, Co‐d/WO_3_, and Co‐d@ZIF‐67/ WO_3_.

**Figure** **2** shows the XRD patterns of Co‐d, WO_3_, Co‐d/WO_3_, Co‐d@ZIF‐67/WO_3_, and Al_2_O_3_ ceramic substrates. The XRD pattern of the in situ grown WO_3_ nanosheets exhibits characteristic peaks corresponding to monoclinic WO_3_ (JCPDS No. 83–0950).^[^ ^34^ ^]^ Specifically, the peaks at 23.21°, 23.34°, 23.42°, 26.75°, 28.94°, 34.19°, and 34.22° correspond to the crystal faces (002), (020), (200), (120), (112), and (022) of WO_3_. This strong match confirms the successful preparation of WO_3_. Similarly, the characteristic peaks of Co‐d align with Co(CO_3_)_0.5_(OH)·0.11H_2_O (JCPDS No. 48–0083).^[^ ^35^, ^36^ ^]^ In the XRD pattern of the Co‐d/WO_3_ material, the peaks at 23.29°, 23.73°, and 24.39° are observed, corresponding to the crystal faces (002), (020), and (200) of monoclinic WO_3_. Additionally, peaks at 26.83° and 28.66°correspond to the (120) and (112) crystal faces of WO_3_. The peaks at 30.61°, 33.32°, and 39.31° represent the (300), (221), and (040) crystal faces of Co‐d. For the Co‐d@ZIF‐67/WO_3_ material, characteristic peaks at 6.8° and 12.5° after the in situ growth of ZIF‐67 were observed, corresponding to the (001) and (112) crystal faces of ZIF‐67.^[^ ^37^ ^]^ Interestingly, as the growth time of ZIF‐67 increases, the peak intensity also rises, indicating an increase in ZIF‐67 content. By controlling the reaction time, the growth of ZIF‐67 material can be effectively regulated. Moreover, the XRD results reveal that the characteristic peaks of WO_3_ material weaken during the layer‐by‐layer growth process. However, the peak positions remain consistent, and no impurity peaks are observed, affirming the successful preparation of Co‐d@ZIF‐67/WO_3_ material.

**Figure 3:**
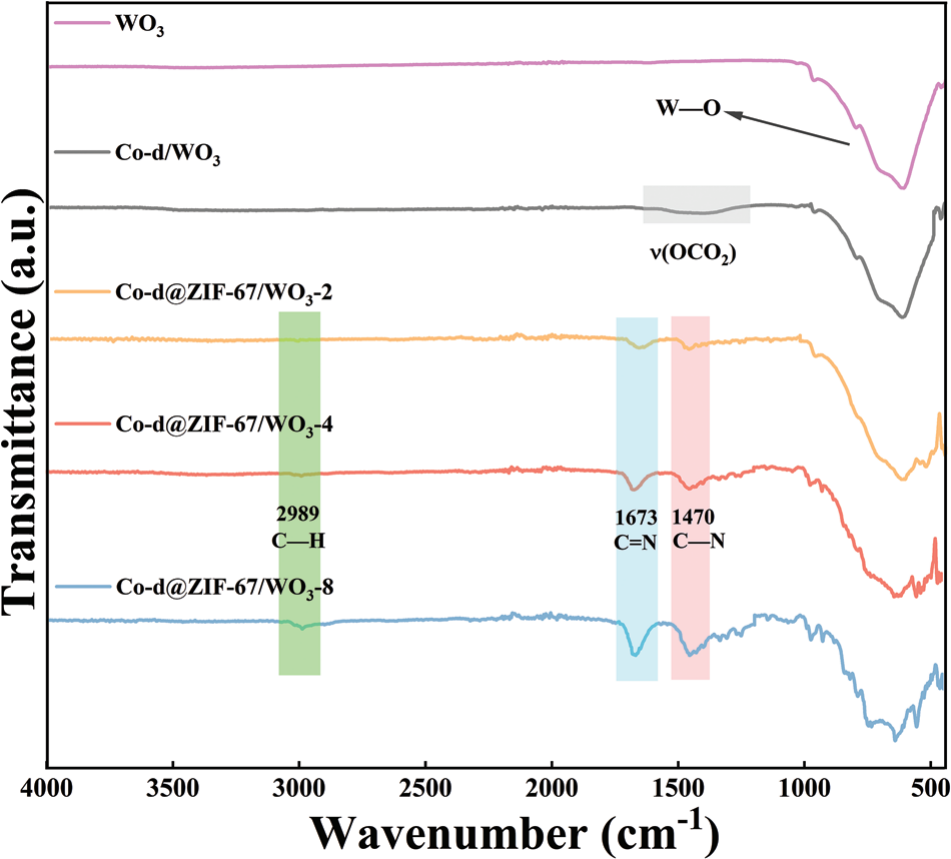
FT‐IR spectra of WO_3_, Co‐d/WO_3_, and Co‐d@ZIF‐67/WO_3_.

To further analyze the structure of the materials, the FT‐IR spectra of WO_3_, Co‐d/WO_3_, and Co‐d@ZIF‐67/WO_3_ are analyzed and illustrated in **Figure** **3**. The absorption band at 629 cm^−1^ in the infrared spectrum of pure WO_3_ material corresponds to the stretching vibration peak of W—O—W,^[^ ^38^ ^]^ supporting the successful preparation of WO_3_. The Co‐d material exhibits absorption bands at 3490 cm^−1^ and 1462 cm^−1^, corresponding to the stretching vibrations of ν(O—H) and ν(OCO_2_), respectively. A spectral band corresponding to ν(C=O) stretching vibration is generated near 892 cm^−1^, while the absorption band at 510 cm^−1^ is attributed to the vibration of ν(Co—O).^[^ ^35^ ^]^ The infrared spectrum of Co‐d/WO_3_ material shows a distinct stretching vibration peak of W—O—W at 629 cm^−1^, along with the appearance of a stretching vibration band at 1462 cm^−1^ corresponding to ν(OCO_2_). The result supports the successful growth of Co‐d material on the surface of WO_3_. After the growth of ZIF‐67 material, characteristic peaks appear at 1470, 1673, and 2989 cm^−1^, corresponding to the stretching vibrations of C—H, C=N, and C—N, respectively.^[^ ^39^ ^]^ This confirms the successful preparation of Co‐d@ZIF‐67/WO_3_ material. The variation in ZIF‐67 peak intensity in Figure 3 corroborates the phenomenon with the increase of the growth time.

**Figure 4:**
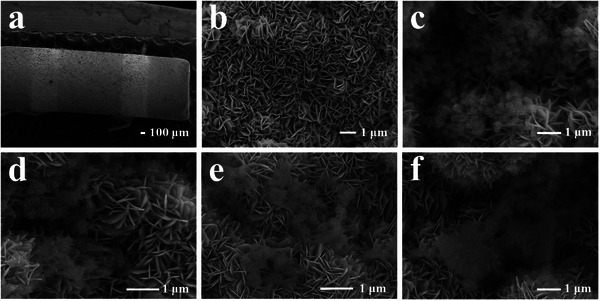
SEM images of a) ceramic tube with in situ grown composite materials, b) WO_3_, c) Co‐d/WO_3_, d) Co‐d@ZIF‐67/WO_3_‐2, e) Co‐d@ZIF‐67/WO_3_‐4 and f) Co‐d@ZIF‐67/WO_3_‐8.

**Figure** **4** shows the FE‐SEM images of different in situ grown materials on the surface of ceramic tubes (Figure 4a). The pure WO_3_ nanosheets are staggered on the ceramic surface to form a dense reticulate substrate structure, with a uniform morphology and distribution, and a thickness of ≈20 nm (Figure 4b). The in situ growth Co‐d material with a sea urchin‐like structure on the WO_3_ substrate surface is shown in Figure 4c. A uniform composite material evenly distributed on the WO_3_ surface was formed with a diameter of ≈400 nm. Subsequently, Co^2+^ were gradually extracted from the surface of the Co‐d material and precipitated through the interaction of the added 2‐methylimidazole. The formation of ZIF‐67 material on the surface is not obvious at the early stage of the reaction (Figure 4d). Over the reaction time, more ZIF‐67 materials can be observed. The Co^2+^ ions in Co‐d are continuously dissolved and bonded with the 2‐methylimidazole ligands. The ZIF‐67 was first grown on the surface of the urchin Co‐d material (Figure 4e). With the increase of Co^2+^ concentration in the solution through the dissolution of Co‐d, the reorganized ZIF‐67 densely accumulates on the WO_3_ surface (Figure 4f). Such dense stacking produces obstacles in gas‐sensing performance.

**Figure 5:**
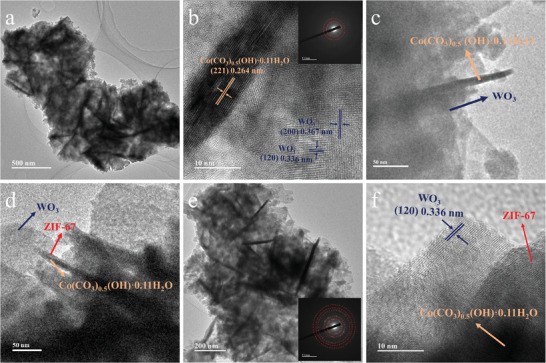
TEM images of a–c) Co‐d/WO_3_ composites and d–f) Co‐d@ZIF‐67/WO_3_ with hierarchical structures.

**Figure 6:**
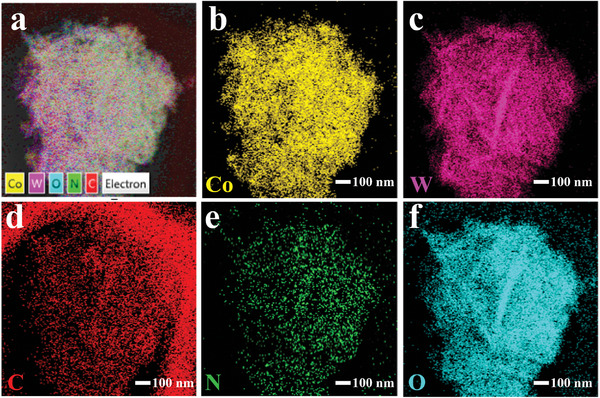
a) EDS images of Co‐d@ZIF‐67/WO_3_‐4 hierarchical structure; b–f) elemental mapping of Co, W, C, N, and O concentrations in the Co‐d@ZIF‐67/WO_3_ hierarchical structure.

To further reveal the surface changes of Co‐d material after the addition of 2‐methylimidazole, the TEM images of the materials before and after the reaction were presented in **Figure** **5**. Figure 5a shows that the Co‐d nanorods, which are the fragmentation of the sea urchin‐like material caused by ultrasonication, tightly interact with the WO_3_ nanosheets. Furthermore, the high‐magnification TEM images in Figure 5b reveal lattice spacings of 0.264 nm, 0.367 nm, and 0.336 nm, which correspond to the (221) lattice of the Co‐d material, the (200) and (120) lattices of monoclinic WO_3_, respectively. These findings further confirm the successful preparation of Co‐d/WO_3_ composite materials. Figure 5c,d reveal that the originally uniform Co‐d nanorod is transformed into a core‐shell structure with the surface coated by ZIF‐67 after adding 2‐methylimidazole. In comparison to Figure 5a,e shows the surface of the Co‐d materials is uniformly coated with ZIF‐67 material. It confirms the formation of ZIF‐67 material, demonstrating the successful preparation of the multi‐level structured Co‐d@ZIF‐67/WO_3_ composite material. **Figure** **6** shows the TEM elemental mapping of Co‐d@ZIF‐67/WO_3_‐4 material. While the Co element is interleaved around WO_3_ nanosheets (Figure 6b), the distribution of the W element presents a clear lamellar structure (Figure 6c). The distribution of the C and N elements in ZIF‐67 material is similar to that of the Co element, but sparser (Figures 6d,e). This is because the formation of the ZIF‐67 epitaxial film relies on the release of Co^2+^ in Co‐d, resulting in the formation of an extremely thin layer only on the surface of Co‐d. Moreover, the distribution of the N element further confirms the synthesis of ZIF‐67 material.

### 3.2 Gas‐Sensing Performance

**Figure 7:**
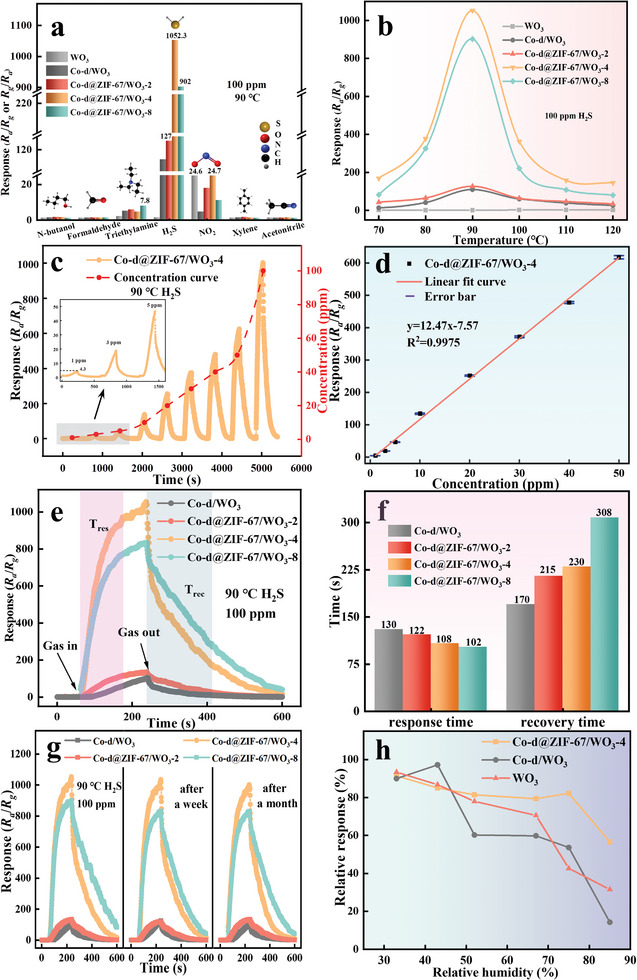
Gas‐sensing performance of WO_3_, Co‐d/WO_3_, Co‐d@ZIF‐67/WO_3_ sensors. a) Selectivity graph; b) Temperature‐sensitivity curves; c) Response curves of Co‐d@ZIF‐67/WO_3_‐4 sensor to different concentrations of H_2_S at 90 °C (The inset shows a magnification at low concentrations); d) Linear relationship between concentration and sensitivity from 1 to 50 ppm H_2_S; e) Dynamic response curves to 100 ppm H_2_S at 90 °C; f) Response/recovery time of different sensors to 100 ppm H_2_S at 90 °C, g) Long‐term stability curves of Co‐d/WO_3_ and Co‐d@ZIF‐67/WO_3_ sensors, h) Humidity stability of different sensors at 90 °C to 100 ppm H_2_S.

The gas‐sensing performance of the sensors fabricated by as‐prepared materials was systematically tested (see **Figure** **7**). Selectivity is an important factor influencing gas‐sensing performance. Its quality directly determines the detection accuracy. As shown in Figure 7a, the different sensors were tested to 100 ppm of various harmful gases (n‐butanol, formaldehyde, triethylamine, H_2_S, NO_2_, xylene, and acetaldehyde) at 90 °C. The results demonstrate that all sensors exhibited good selectivity to H_2_S except for the pure WO_3_ sensor. The excellent selectivity of the composite material can be ascribed to the following factors: (1) compared with other volatile organic compounds, H_2_S has a relatively small bond dissociation energy (381 kJ/mol),^[^ ^40^ ^]^ which is beneficial for the decomposition and surface reaction in the chemical adsorption process at low temperatures; (2) on the same surface adsorption area, H_2_S is considered to have a larger diffusion rate through the pore of ZIF‐67 due to its smaller molecular size among these gas molecules;^[^ ^41^ ^]^ and (3) the pentagonal N heterocycle in the ZIF‐67 material has special adsorption properties for H_2_S because of the stronger affinity of electron‐rich N atoms with acidic H_2_S.^[^ ^42^ ^]^

With the increase in the variety of in situ grown materials on the surface of WO_3_, the response to H_2_S gradually increased. The response value of the Co‐d@ZIF‐67/WO_3_‐4 to 100 ppm H_2_S at 90 °C reaches 1052.3, which is 779 times higher than that of pure WO_3_ sensors and 9.36 times higher than that of Co‐d/WO_3_ sensors. The response values of the Co‐d@ZIF‐67/WO_3_ sensors increase during the ZIF‐67 growth time, reaching the maximum value when the ZIF‐67 growth time is 4 h. However, the Co‐d@ZIF‐67/WO_3_‐8 with a longer ZIF‐67 growth time has an overly dense accumulation of ZIF‐67 material from FE‐SEM, as suggested in Figure 4f, which affects the gas transfer process. As such, the degradation of sensing performance is observed.

The operating temperature exerts a crucial influence on the performance, operating conditions, and lifespan of gas sensors. Figure 7b demonstrates that the response values of the other sensors to H_2_S exhibit an initial increase followed by a decrease except for the WO_3_ sensors. It can be attributed to the impact of temperature on the adsorption and desorption processes of gases on the surface of materials. At lower temperatures, the activation energy results in a weak reaction extent of the adsorbed gases. When the temperature rises, the provided energy prompts the reaction between the adsorbed gases and surface oxygen molecules, which leads to a more pronounced change in surface resistance and an increase in the sensor response. However, the excessive energy on the sensor surface at higher temperatures causes the desorption of adsorbed gas molecules, which reduces available gas molecules for surface reactions and decreases the response. The in situ growth of Co‐d facilitates the formation of surface heterojunction structures, while the catalytic effect of Co^2+^ on the surface reduces the activation energy for the reaction between adsorbed analytes and oxygen molecules. Consequently, the material achieves a balance between adsorption and desorption at lower temperatures, resulting in a lower operating temperature for the sensors. Additionally, the inclusion of porous ZIF‐67 materials provides a larger adsorption capacity for gas molecules to enhance the sensor response.

To investigate the response of the Co‐d@ZIF‐67/WO_3_‐4 sensors to low concentrations of H_2_S and their LOD, several gas‐sensing tests were performed on the Co‐d@ZIF‐67/WO_3_‐4 sensor in the range of 1–100 ppm. The results are shown in Figure 7c. As the concentration increases, the response value of the sensors also continuously increases. Even at the low concentration of 1 ppm, a good response curve was obtained, with a response value as high as 4.3. The LOD value of the sensors was determined by plotting the concentration‐response value curve from 1 to 50 ppm. Figure 7d illustrates the linear fitted curve of concentration and sensitivity. The correlation coefficient R^2^ of the fitting curve in the range of 1 − 50 ppm is 0.9975, which indicates excellent linearity between the response and H_2_S gas concentration. The slope of the fitted curve is 12.47. Therefore, the LOD of the sensor based on Co‐d@ZIF‐67/WO_3_‐4 sensor is 16.72 ppb according to equation (1), which shows that the sensor has great application potential in the field of ultra‐low H_2_S concentration detection.

The response/recovery time of the sensor is an important parameter in its real‐time application. The fabricated components were tested for response/recovery time. The results are shown in Figure 7e,f. By controlling the same injection and exhaust time, the time taken for the device to reach 90% of the maximum change was measured. Figure 7e shows that as the growth time of ZIF‐67 material increased, the response time of the material was gradually shortened, while the recovery time increased. The results may be attributed to the unique adsorption characteristics of ZIF‐67 material while forming a chemical reaction with H_2_S,^[^ ^43^ ^]^ which makes it easier to adsorb H_2_S molecules onto its surface. Furthermore, the trend becomes more pronounced with an increase of the ZIF‐67 growth time. The prolongation of the recovery time is due to the difficult desorption of the strongly adsorbed species combined with the rich porous structure of the ZIF‐67 material. At the early stage of the reaction (growth time of 2 h), the ZIF‐67 was not fully formed with short internal pore channels. As the growth proceeded, the internal pores of ZIF‐67 were continuously extended (growth time of 4 and 8 h), which made the desorption path of H_2_S molecules longer. Channel structure diagrams for ZIF‐67 with different reaction time are shown in Figure S3 (Supporting Information). Consequently, its desorption time is prolonged. Overall, the Co‐d@ZIF‐67/WO_3_‐4 device exhibits a short response/recovery time (108 s/ 230 s), making it suitable for the practical detection of H_2_S.

The stability of a component is directly related to its service life. The stability test results are shown in Figure 7g. It can be found that the sensing performance at 90 °C to 100 ppm H_2_S is almost identical after one week and one month, which demonstrates the good stability and repeatability of the device. The gas‐sensing performance of WO_3_, Co‐d/WO_3_, and Co‐d@ZIF67/WO_3_‐4 is shown in Figure 7h under different relative humidity conditions (33%, 43%, 52%, 67%, 75%, 85%). After the in situ growth of Co‐d on the WO_3_ surface, its humidity immunity significantly decreased although the response value of the material increased. It may be due to the presence of hydrophilic groups (OH) in the Co‐d material,^[^ ^44^ ^]^ resulting in a decrease in performance in high‐humidity environments. However, after the in situ growth of ZIF‐67 outside the Co‐d material, the Co‐d@ZIF67/WO_3_‐4 device exhibited excellent humidity immunity, maintaining 82.2% response value in ambient air under a high humidity condition (75%RH). In the ZIF‐67 material, the methyl groups on the organic ligands point to the interior of the pores and the internal pores are highly hydrophobic.^[^ ^45^, ^46^ ^]^ As shown in Figure S4 (Supporting Information), the contact angles of WO_3_, Co‐d/WO_3_, Co‐d@ZIF‐67‐2/WO_3_, Co‐d@ZIF‐67‐4/WO_3_ and Co‐d@ZIF‐67‐8/WO_3_ nanomaterials are 38.59°, 29.21° 63.59°, 86.81° and 104.3°, respectively. After combining with Co‐d materials, the composite became hydrophilic due to the hydrophilic groups contained in Co‐d/WO_3_ materials. The hydrophobicity is significantly improved by forming ZIF‐67 epitaxial film on the surface of the Co‐d/WO_3_ material. The contact angles verify that the strategy of the introduction of hydrophobic ZIF‐67 film is feasible for improving the humidity immunity of gas sensors. The gas‐sensing performance of different H_2_S sensors in recent years is shown in **Table** **1**.

**Table 1:** Comparison of gas‐sensing performance of different H_2_S gas sensors.

Materials	Operating temperature (°C)	Concentration (ppm)	Response/recovery time (s)	Response *R_a_*/*R_g_* or (*R_a_*‐*R_g_*)/*R_g_* ^a)^	Limit of detection	Relative response/(relative humidity)	Ref.
ZnO/ZnCo_2_O_4_	160	100	100/94^b)^	65.38	30 ppb	90%/ (85%)	[47]
MoO_3_/CuO/g‐C_3_N_4_	RT	1	49/210	8.24	50 ppb	–	[48]
MoS_2_‐GO	RT	100	6.1/302	39.16^a)^	1 ppm	18%/ (80%)	[49]
α‐Fe_2_O_3_/SnO_2_	250	10	14/105	4.3	2 ppm	–	[50]
PdO/ZnO/ZnS	100	10	174/102	212.4	1 ppm	25%/ (59%)	[51]
In_2_O_3_/ZnO	200	50	52/198	67.5	600 ppb	–	[52]
SnSe_2_/WO_3_	25	10	136/459	33.8^a)^	10 ppm	33.8%/ (82%)	[53]
Co‐d@ZIF‐67/WO_3_	90	100	108/230	1052.3	7.26 ppb	82.2%/ (75%)	This work

^a)^ Response value calculated in (*R_a_*‐*R_g_*)/*R_g_*.;

^b)^ Incomplete recovery.

### 3.3 Gas‐Sensing Mechanism

During the gas‐sensing testing procedure, oxygen molecules in the air are adsorbed onto the surface of composite materials, capturing electrons from the sensors and generating chemisorbed oxygen species such as O^−^, O_2_^−^, and O^2−^. Consequently, a depletion layer is formed on the surface of the composite materials.^[^ ^54^ ^]^ The type of adsorbed oxygen species relies on the operating temperature. When temperatures are below 100 °C, chemisorbed oxygen typically adopts the form of O_2_^−^ on the surface of the material. Between 100 and 300 °C, O_2_^−^ rapidly converts to O^−^ on the surface. Above 300 °C, O^2‐^ becomes the predominant oxygen species.^[^ ^55^ ^]^ Given the sensing tests were conducted at 90 °C in this study, O_2_^−^ is commonly chemisorbed on the surface. The detailed reaction process can be described as follows:

(2) $$O_{2(g)}\to O_{2(\mathrm{ads})}$$

(3) $$O_{2\left(\mathrm{ads}\right)}+e^{-}\to O_{2(\mathrm{ads})}^{-}$$

The subscripts (g) and (ads) in Equation (2) and (3) respectively denote oxygen molecules as gaseous species and as molecules adsorbed on the surface. Upon exposure to H_2_S environment, H_2_S molecules are transferred to the surface of the composite materials and then adhere to it. Subsequently, these molecules react with previously adsorbed O_2_^−^, leading to the production of SO_2_ and H_2_O.^[^ ^47^, ^56^ ^]^ Throughout this reaction process, the captured electrons are liberated and returned to the sensing materials, resulting in a reduction in the electronic depletion layer, an elevation in carrier concentration, and a decrease in the resistance of the material.

**Figure 8:**
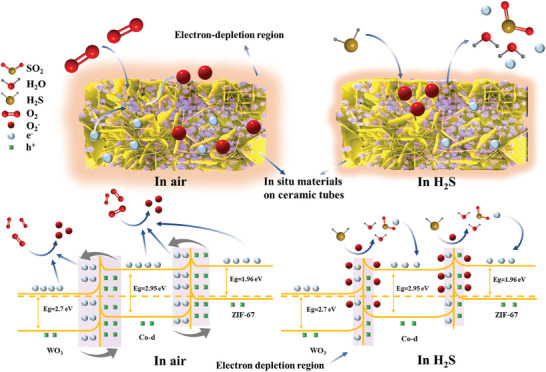
Schematic diagram of the energy band and sensing mechanism for Co(CO_3_)_0.5_(OH)·0.11H_2_O@ZIF‐67/WO_3_ in air and H_2_S.

The enhanced gas‐sensing performance of the material is primarily due to the change of energy band structure resulting from the interaction of different materials.^[^ ^57^ ^]^ The energy band diagram portraying the Co‐d@ZIF‐67/WO_3_ material is depicted in **Figure** **8**. In ambient air, the energy levels of the conduction band and valence band for WO_3_, Co‐d, and ZIF‐67 are ≈0.74 and 3.44 eV, −0.78 and 2.17 eV, and −1.05 and 0.91 eV, respectively.^[^ ^26^, ^58^, ^59^ ^]^ When the WO_3_ material contacts with Co‐d in the air, charge transfer transpires at their interface due to the difference in the Fermi energy levels of the materials. Electrons are transferred from Co‐d to WO_3_ until the Fermi level attains equilibrium, inducing the formation of an interface barrier between WO_3_ and Co‐d. Consequently, the depletion region at the interface enlarges with the enhanced gas‐sensing performance. Subsequently, as the ZIF‐67 material is grown on the Co‐d surface, electrons are transferred from the ZIF‐67 material to the Co‐d material. Once the Fermi level reaches equilibrium, the height of the heterojunction barrier further escalates, leading to the expansion of the space depletion region at the interface and the consequent enhancement of gas‐sensing performance. Upon exposure of the composite materials to H_2_S, the reaction between H_2_S and the adsorbed oxygen molecules (O_2_^−^) on the surface of materials diminishes the electronic depletion layer at the interface, resulting in a reduced resistance of materials.

**Figure 9:**
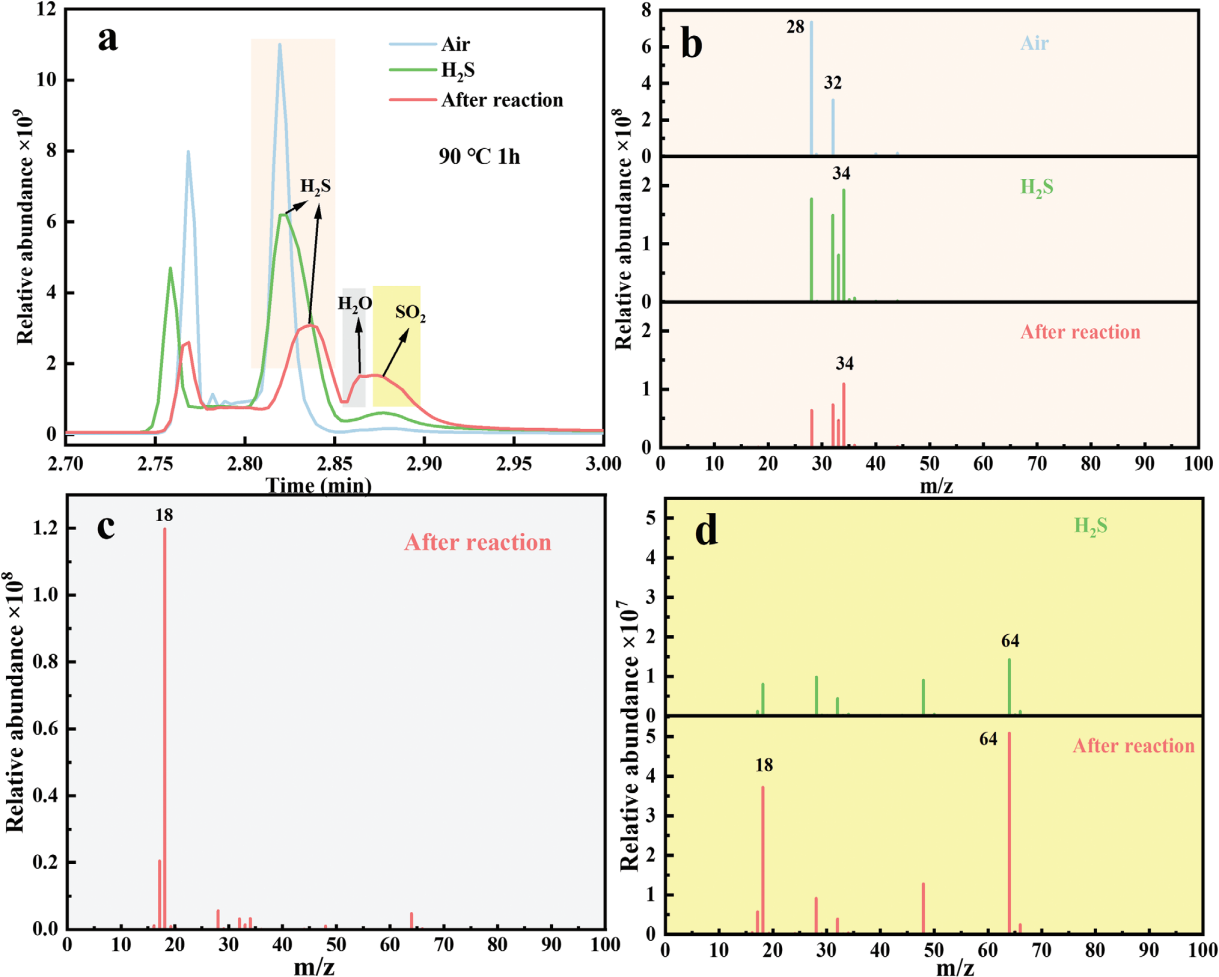
Gas chromatograms a) and mass spectra b–d) of H_2_S and gas products before or after reaction of Co‐d@ZIF‐67/WO_3_‐4 composites with H_2_S.

To confirm this reaction mechanism, the background and the atmosphere before and after the reaction were characterized by GC‐MS as illustrated in **Figure** **9**. Figure 9a shows the GC diagrams of gases in the headspace vials after reacting with air, H_2_S, and Co‐d@ZIF‐67/WO_3_‐4 composite material at 90 °C for 1 h. New peaks appeared at 2.86 min and 2.88 min after the samples reacted with H_2_S. To identify corresponding substances at different retention time, MS analyses were conducted except for the peak at 2.75‐2.78 min (N_2_ in the air). Figure 9b exhibits the MS diagrams of different materials within 2.80–2.85 min. The mass charge ratios (m/z) of 28 and 32 in the air MS diagram mainly correspond to N_2_ and O_2_, respectively, while m/z of 34 in the H_2_S and after reaction MS corresponds to H_2_S. Figure 9c shows the MS diagram corresponding to the peak at 2.86 min after the reaction, where the peak of m/z = 18 demonstrates that the H_2_O is produced. Figure 9d compares the MS of H_2_S and the gas after reaction at 2.88 min, which reveals that the SO_2_ signal (m/z = 64) has significantly increased (a small amount of SO_2_ is contained in the H_2_S gas, which is probably due to oxidation of H_2_S in the air). Our GC‐MS results, therefore, prove that the reaction products of Co‐d@ZIF‐67/WO_3_‐4 composite material with H_2_S at the optimal working temperature are SO_2_ and H_2_O, which confirms the proposed mechanism. By comparing the peak intensity change, the stoichiometry of the reaction can be roughly inferred as below:

(4) $$2H_{2}{S+3O}_{2}^{-}\to 2H_{2}O+2SO_{2}+3e^{-}$$

## 4 Conclusion

In this study, ternary Co(CO_3_)_0.5_(OH)∙0.11H_2_O@ZIF‐67/WO_3_ composite material with hierarchical structures was in situ synthesized using a layer‐by‐layer growth method. Co‐d@ZIF‐67/WO_3_‐4 sensors with superior H_2_S gas‐sensing performance were obtained by carefully controlling the ZIF‐67 growth time at 4 h. Their gas‐sensing test results indicated a remarkable response value of 1052.3 (779 times higher than that of pure WO_3_ and 9.36 times higher than that of Co‐d/WO_3_) to 100 ppm H_2_S at a low operating temperature of 90 °C. It also showed a short response/recovery time (108 s/230 s), good repeatability, and long‐term stability, with only an 18% decrease in response value under a high humidity condition (75% RH). The excellent gas‐sensing performance can be attributed to the heterojunction structure, synergistic effect between WO_3_, Co(CO_3_)_0.5_(OH)∙0.11H_2_O and ZIF‐67, and humidity immunity properties of the ZIF‐67 material. (1) The composite of WO_3_, Co‐d, and ZIF‐67 leads to the formation of a multilayered heterojunction structure and induced modification of the surface of the energy band structure of the materials. (2) The presence of the ZIF‐67 material with its distinctive pentagonal N heterocycle provides a porous structure that facilitates a unique adsorption effect on H_2_S which enhances the response and selectivity of the gas sensor. (3) The humidity immunity exhibited by the ZIF‐67 material grown from Co‐d ensures excellent stability of the composite materials even under high humidity conditions. As a result, the construction of the hierarchical material significantly enhances the response value, lowers the operating temperature, and improves stability under high humidity conditions. Our results demonstrate that the in situ method through a layer‐by‐layer growth of the composite material offers a new approach to fabricate gas‐sensing elements and allows for the development of diverse WO_3_‐based gas‐sensing materials. This method has significant applications in environmental monitoring and smart healthcare.

## Conflict of Interest

The authors declare no conflict of interest.

## Author Contributions

Y.G. performed conceptualization, resources, funding acquisition, writing – review & editing. J.W. performed conceptualization, investigation, methodology, and writing – original draft. D.Z. performed software, methodology & validation. K.T. performed writing – original draft, conceptualization. S.Z. performed writing – review & editing. H.G. performed writing – review & editing, and funding acquisition. X.Q. performed conceptualization, and funding acquisition. X.Q. performed writing – review & editing, funding acquisition. D.G. performed writing – review & editing. Y.W. performed resources, writing – review & editing, and funding acquisition.

## Supporting information

Supporting Information

## Data Availability

The data that support the findings of this study are available from the corresponding author upon reasonable request.
