# Supplementary material for: In Situ Synthesis of Hierarchical Co(CO3)0.5(OH)·0.11H2O@ZIF‐67/WO3 With High Humidity Immunity and Response to H2S Sensing
Source: Adv Sci (Weinh). 2024 Aug 19;11(39):2402352. doi: 10.1002/advs.202402352 (PMC11497035; doi:10.1002/advs.202402352)
Supplement: Supplementary file 1 — Supporting Information [file ADVS-11-2402352-s001.docx]

**Supporting information**

**In-Situ Synthesis of Hierarchical Co(CO_3_)_0.5_(OH)·0.11H_2_O@ZIF-67/WO_3_ with High Humidity Immunity and Response to H_2_S Sensing**

*Yanghai Gui*, Jintao Wu, Di Zhao, Kuan Tian, Shuaishuai Zhao, Huishi Guo, Xiaoyun Qin, Xiaomei Qin, Dongjie Guo, Yun Wang**

*Y. Gui, J. Wu, K. Tian, S. Zhao, H. Guo, X. Qin, X. Qin, D. Guo,*

*College of Materials and Chemical Engineering*

*Zhengzhou University of Light Industry*

*Zhengzhou 450000, China*

*E-mail: yhgui@zzuli.edu.cn*

*D. Zhao, Y. Wang*

*Centre for Catalysis and Clean Energy, School of Environment and Science*

*Griffith University*

*Gold Coast, QLD 4222, Australia*

*E-mail: yun.wang@griffith.edu.au*

**Statistical Analysis details:**

The limit of detection (LOD) was calculated based on the following equations.

$\text{LOD=3×σ/S}$ (1)

$\text{σ(r)=}\sqrt{\frac{\text{1}}{\text{N}}\sum_{\text{i=1}}^{\text{N}} {\text{(}\text{X}_{\text{i}}\text{-r)}}^{\text{2}}}$ (2)

where σ is the standard deviation, which represents the root mean square noise of the baseline. S is the slope of the fitted concentration-response linear curve. Herein, the σ is calculated as 0.069511 based on 60 data points of the baseline curve of Co-d@ZIF-67/WO_3_-4 sensor. The 60 baseline resistance data were collected at each second of one minute, which are listed in **Table S1 (N=60).**

**Table S1.** The baseline resistance of the Co-d@ZIF-67/WO_3_-4 sensor

| Time (s) | Resistance (Ω) | Time (s) | Resistance (Ω) | Time (s) | Resistance (Ω) |
| --- | --- | --- | --- | --- | --- |
| 1 | 4020000 | 21 | 3990564 | 41 | 4179188 |
| 2 | 4067104 | 22 | 3990564 | 42 | 4229085 |
| 3 | 4020841 | 23 | 3990564 | 43 | 4229085 |
| 4 | 4020841 | 24 | 4020841 | 44 | 4229085 |
| 5 | 4067104 | 25 | 3990564 | 45 | 4229085 |
| 6 | 4067104 | 26 | 4020841 | 46 | 4229085 |
| 7 | 4067104 | 27 | 4020841 | 47 | 4229085 |
| 8 | 4098526 | 28 | 4020841 | 48 | 4179188 |
| 9 | 4098526 | 29 | 4020841 | 49 | 4179188 |
| 10 | 4067104 | 30 | 4067104 | 50 | 4146554 |
| 11 | 4067104 | 31 | 4067104 | 51 | 4146554 |
| 12 | 4020000 | 32 | 4067104 | 52 | 4146554 |
| 13 | 4067104 | 33 | 4067104 | 53 | 4146554 |
| 14 | 4020841 | 34 | 4098526 | 54 | 4098526 |
| 15 | 4020841 | 35 | 4098526 | 55 | 4146554 |
| 16 | 4020841 | 36 | 4146554 | 56 | 4146554 |
| 17 | 3990564 | 37 | 4098526 | 57 | 4098526 |
| 18 | 4020841 | 38 | 4098526 | 58 | 4098526 |
| 19 | 4020841 | 39 | 4098526 | 59 | 4098526 |
| 20 | 4020841 | 40 | 4098526 | 60 | 4098526 |


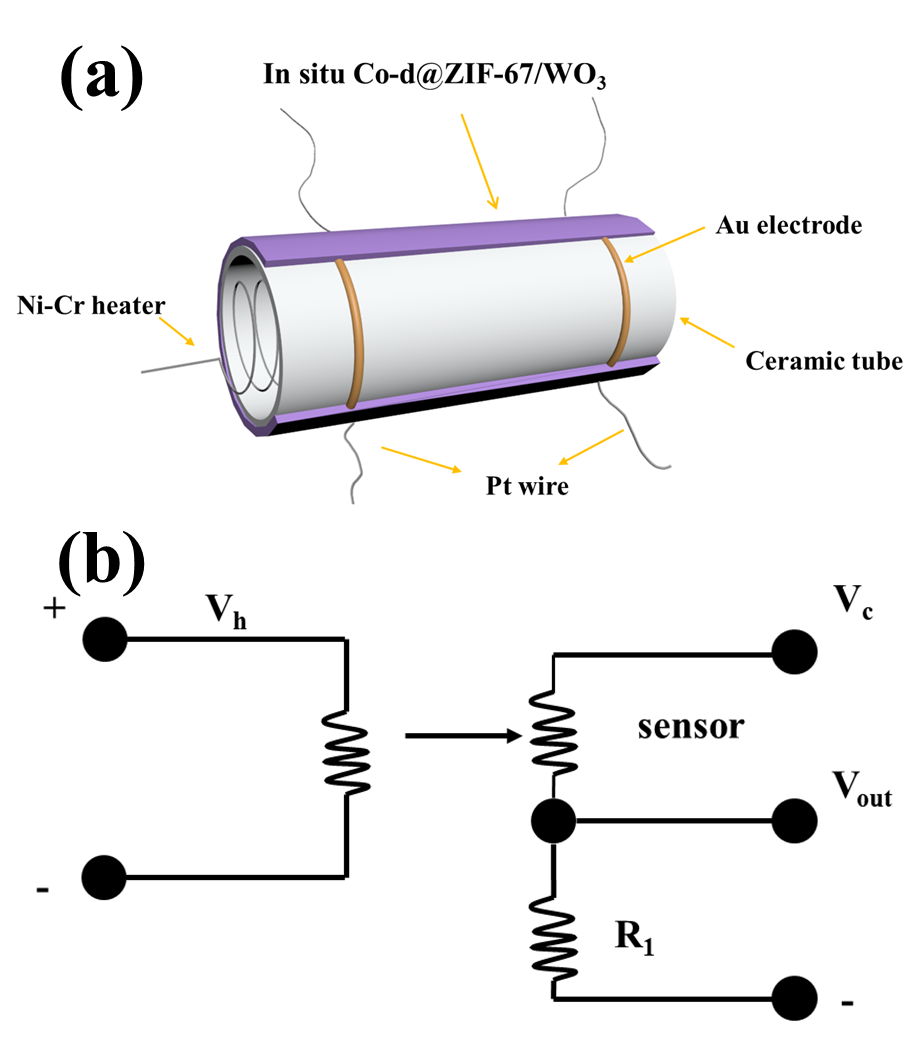


**Figure S1.** The diagrams of gas sensor structure (a) and test system circuit (b).

The power supply for the working heating of the gas-sensing sensor is denoted as V_h_, while V_c_ represents the loop power supply (in **Figure. S1**). By measuring the voltage (V_out_) across the load resistor R_1_, which is connected in series with the gas sensor, the characteristics of the gas sensor are ascertained.


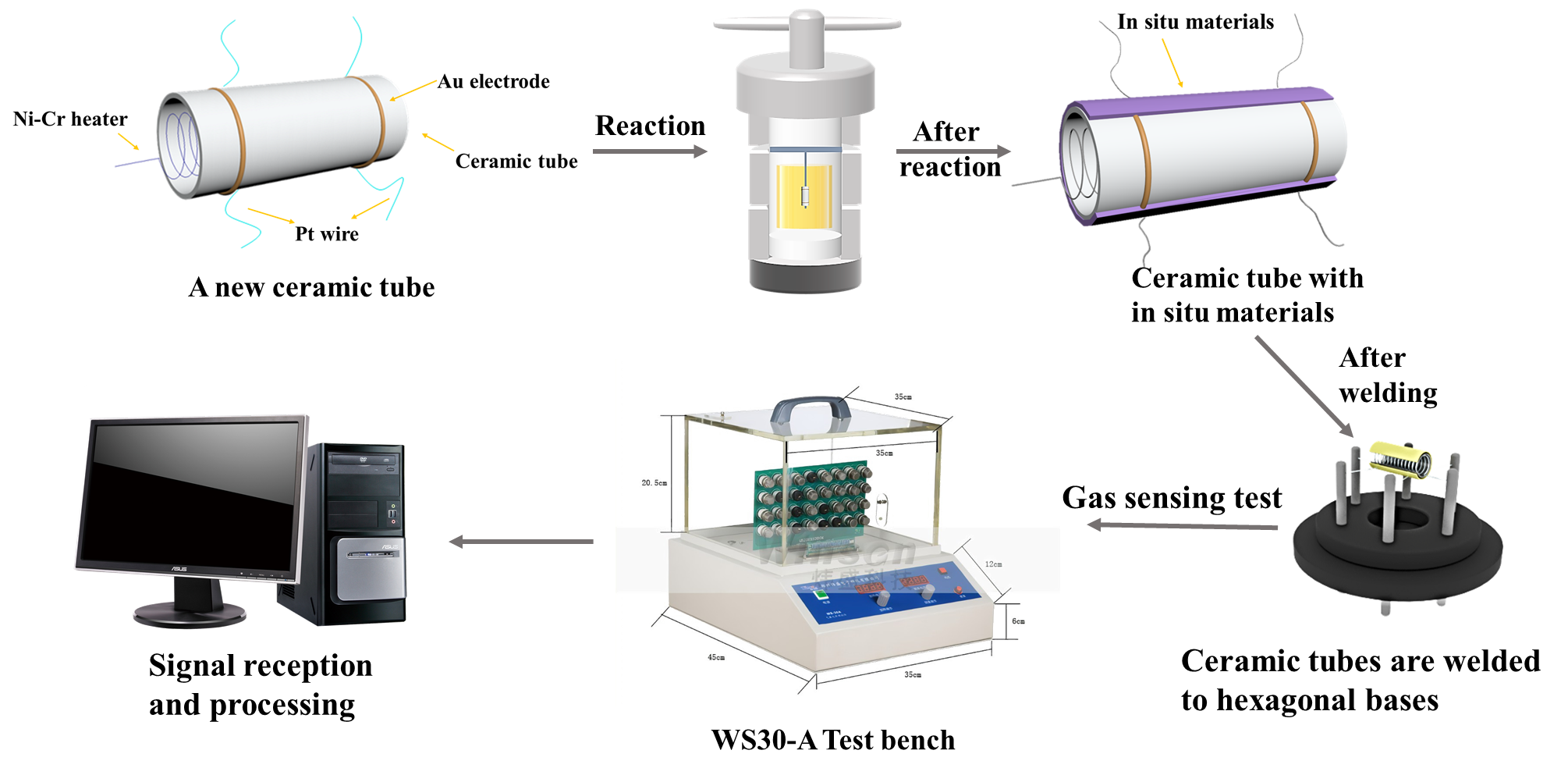


**Figure S2.** Schematic of preparation and testing process of gas sensors


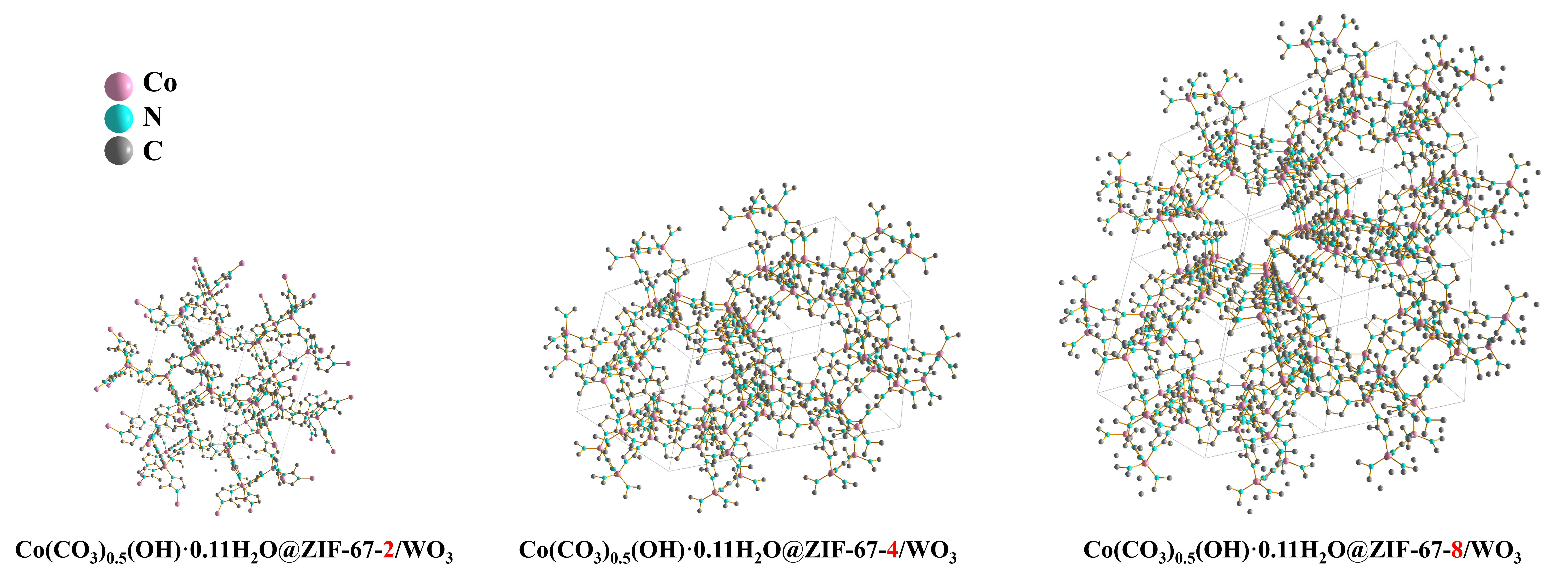


**Figure S3.** Crystal structure of ZIF-67 on the surface of Co(CO_3_)_0.5_(OH)·0.11H_2_O@ZIF-67/WO_3_

**
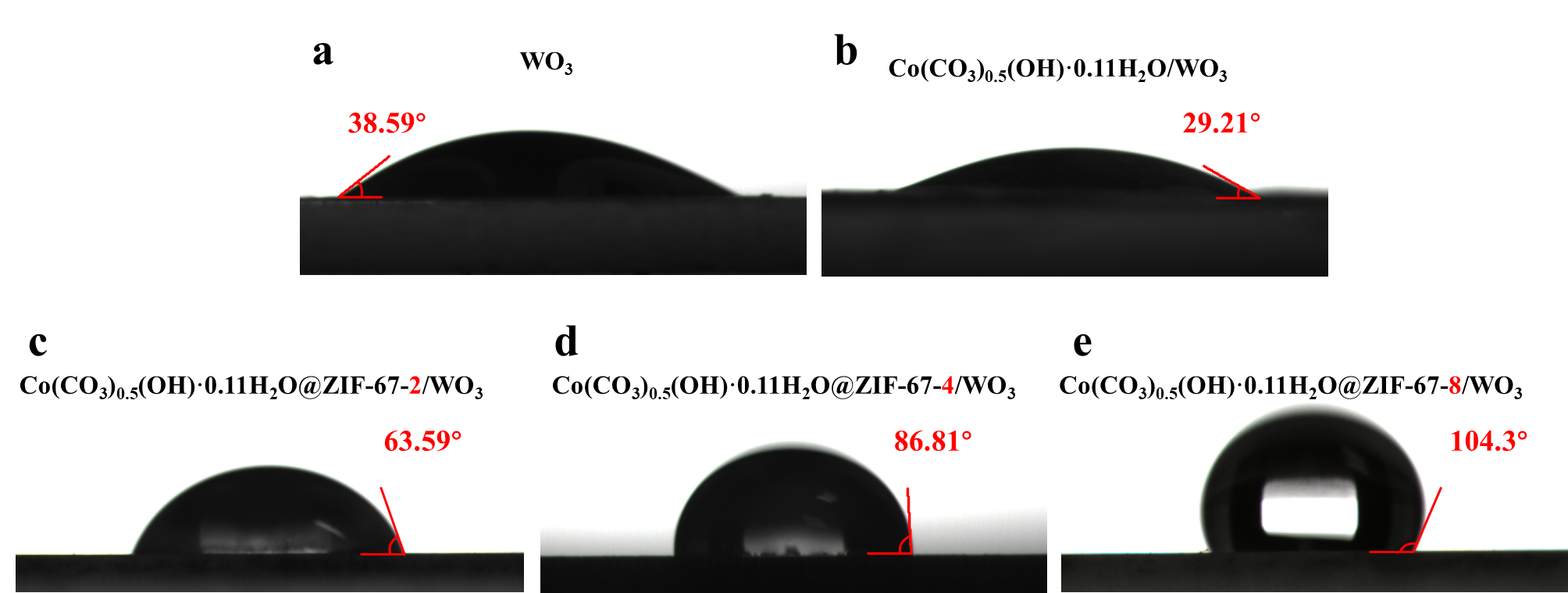
**

**Figure S4.** Contact angles of (a) WO_3_, (b) Co(CO_3_)_0.5_(OH)·0.11H_2_O/WO_3_, and (c, d and e) Co(CO_3_)_0.5_(OH)·0.11H_2_O@ZIF-67/WO_3_ materials.

As shown in **Figure. S4**, the contact angles of WO_3_ and Co(CO_3_)_0.5_(OH)·0.11H_2_O/WO_3_ nanomaterials are 38.59° and 29.21°. After combining with Co(CO_3_)_0.5_(OH)·0.11H_2_O materials, the composite became hydrophilic due to the hydrophilic groups contained in Co(CO_3_)_0.5_(OH)·0.11H_2_O/WO_3_ materials. Depending on the reaction time, the hydrophobicity of the materials appeared to be significantly different, with contact angles of 63.59, 86.81, and 104.3 for the materials with reaction times of 2, 4, and 8 h. The hydrophobicity is significantly improved by forming ZIF-67 epitaxial films on the surface of the Co(CO_3_)_0.5_(OH)·0.11H_2_O/WO_3_ material. Based on the Young equation, the surface energies of WO_3_, Co-d/WO_3_, Co-d@ZIF-67/WO_3_-2, Co-d@ZIF-67/WO_3_-4 and Co-d@ZIF-67/WO_3_-8 are estimated to be 129.70 mN/m, 136.34 mN/m, 105.18 mN/m, 76.8 mN/m, and 54.8 mN/m, respectively.
